# Correlation Analysis Between Immune Status and Bronchoscopic Airway Findings in Various Ages of SMPP Children

**DOI:** 10.1155/carj/6145605

**Published:** 2026-06-03

**Authors:** Peng Li, Ruiyang Sun, Jiapu Hou, Zewen Ding, Wanyu Jia, Shuqin Fu, Junhao Cui, Chunlan Song

**Affiliations:** ^1^ Department of Emergency Medicine, Children’s Hospital Affiliated to Zhengzhou University, Henan Children’s Hospital Zhengzhou Children’s Hospital, Zhengzhou, Henan, China, zzusah.com

**Keywords:** bronchoalveolar lavage, immunoglobulin, mucosal erosion, severe *Mycoplasma pneumoniae* pneumonia

## Abstract

**Objective:**

To investigate the correlation between trends in immune markers and airway mucosal changes in SMPP by controlling for age.

**Study Design:**

A total of 302 children with SMPP admitted to Henan Children’s Hospital from 2018 to 2024 were selected as the study subjects for retrospective analysis, while 133 children with GMPP were selected as the control group. The children were divided into infancy groups, toddler groups, preschool age groups, and school age groups according to their ages.

**Results:**

(1) There were significant differences in some clinical manifestations and laboratory hematological indices among SMPP children of various ages. (2) In the SMPP group, compared to toddlers and preschool children, school‐aged children had a higher rate of mucosal erosion, and compared to infants, school‐aged children had a higher rate of mucous plug blockage (*p* < 0.05). (3) Pearson correlation analysis revealed that IgM was positively correlated with mucosal erosion and mucous plug plugging, the AGR was negatively correlated with mucosal erosion and mucous plug plugging, and the NLR and D‐D were positively correlated with mucous plug plugging in preschool‐aged and school‐aged children. IgA and FIB were positively correlated with mucous plug blockage, and FIB was positively correlated with mucosal erosion in school‐aged children with SMPP (*p* < 0.05).

**Conclusion:**

IgM, IgA, NLR, AGR, FIB, and D‐D in preschool‐aged and school‐aged children with MP infection are potential markers of disease severity and the degree of airway mucosal damage.

## 1. Introduction

*Mycoplasma pneumoniae* pneumonia (MPP) is a community‐acquired pneumonia that is more common in children aged 5 years and above. Its pathogenesis may be related to the direct invasion of *Mycoplasma pneumoniae* (MP) into epithelial cells and the immune imbalance of the body [1]. MPP is generally considered to be a mild, self‐limiting disease, but mortality and complication rates have increased in recent years [2], with approximately 12% of MPPs progressing to severe MPP (SMPP) [3]. SMPP can lead to severe pulmonary lesions, such as pleural effusion, pulmonary consolidation, pulmonary embolism, and even pulmonary necrosis. It also involves systems other than the respiratory system, such as circulation, digestion, blood, skin, and mucosa [4].

Some studies suggest that compared to other pathogens, MPP causes more serious airway mucosal damage. In the acute stage of MPP, airway mucosa may show different degrees of damage under bronchoalveolar lavage (BAL), such as mucosal hyperemia and swelling, mucosal erosion, mucous plug blockage of the lumen, and inflammatory stenosis of the lobe or segmental bronchial opening [5]. There are significant differences in chest imaging and clinical symptoms among children with MPP at various ages, but there are no relevant reports on whether there are differences in the degree of airway damage under bronchoscopy in children with SMPP at various ages.

The purpose of this study was to retrospectively analyze the clinical symptoms, immune function, and airway characteristics of SMPP children at various ages who underwent BAL. First, the characteristics of airway damage during bronchoscopy in children with SMPP at various ages were investigated. Second, we explored the correlation between the change in immune levels in children with SMPP at various ages and different airway manifestations under bronchoscopy to provide some clinical indicators for the early diagnosis and treatment of SMPP.

## 2. Data and Methods

Patients: This was a retrospective study. A total of 302 children with SMPP and 133 children with generalized MPP (GMPP) hospitalized at Henan Provincial Children’s Hospital from 2018 to 2024 were selected as the study objects. The laboratory test results and clinical data were extracted from the hospital’s electronic medical records system. This study was approved by the Medical Ethics Committee of Henan Children’s Hospital (2025‐KY‐0037‐001).

The subjects were grouped according to age: 0–12 months in the infant group, 13–36 months in the toddler group, 37–72 months in the preschool age group, and 73–168 months in the school age group.

The diagnostic criteria for MPP are as follows [6]: (1) clinical manifestations such as fever, cough, asthma, and dyspnea; (2) abnormal chest imaging findings; and (3) one or more of the following criteria are met: serum MP antibody titer ≥ 1:160, double the serum MP antibody titer ≥ 4 times, or MP‐DNA and/or MP‐RNA positive.

SMPP meets one or more conditions: (1) temperatures persist above 39°C for ≥ 5 days or fever for ≥ 7 days. (2) Clinical symptoms such as wheezing, dyspnea, chest pain, and hemoptysis. (3) Extrapulmonary complications, such as those involving the digestive system and cardiovascular system. (4) Transcutaneous oxygen saturation ≤ 93%. (5) Imaging findings ≥ 1 condition: a single lung lobe ≥ 2/3 involved or two or more lung lobes had dense solid lesions, which may have been accompanied by pleural effusion and localized bronchiolitis symptoms. Single lungs diffuse or bilateral ≥ 4/5 of the lungs with bronchiolitis symptoms and atelectasis. (6) The range of imaging lesions progressed rapidly > 50% within 1–2 days. (7) C‐reactive protein (CRP), lactate dehydrogenase (LDH), and D‐dimer (D‐D) levels were abnormally elevated. Children with no above performance were categorized into the GMPP group.

The inclusion criteria for patients were as follows: aged 0–14 years, met the SMPP diagnostic criteria, and had complete clinical data.

**FIGURE 1:**
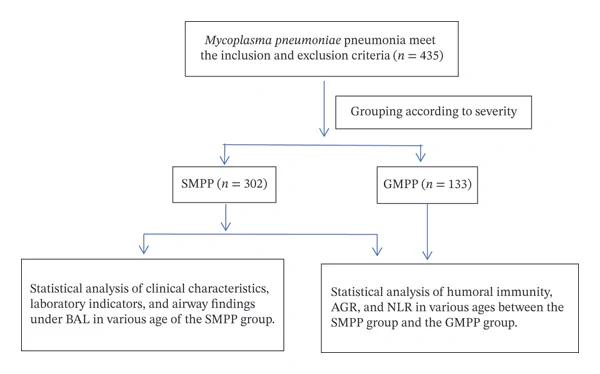
Flowchart.

The exclusion criteria were as follows: (1) underlying immunodeficiency, (2) congenital malformations of the respiratory system, (3) prior steroid/immunoglobulin therapy, (4) chronic pulmonary disease, (5) referral from malignant tumors, (6) incomplete clinical data, and ≤ 14 years of age (Figure 1).

## 3. Clinical Data Collection

Collected clinical data were divided into five categories: ①General information, ②clinical presentation, ③airway characteristics under a BAL microscope (diagnosis is carried out through standardized criteria), ④the presence of other pathogens, ⑤the laboratory data from the first test after admission included white blood cell (WBC) count, platelet (PLT) count, CRP, Interleukin‐6 (IL‐6), procalcitonin (PCT), Immunoglobulin G (IgG), Immunoglobulin A (IgA), Immunoglobulin M (IgM), Complement C3 (C3), Complement C4 (C4), creatinine (CR), urea (Urea), uric acid (UA), creatine kinase (CK), CK isoenzyme (CK‐MB), erythrocyte sedimentation rate (ESR), prothrombin time (PT), partially activated PT (APTT), fibrinogen (FIB), D‐D, total protein (TP), alanine aminotransferase (ALT), aspartate aminotransferase (AST), and LDH.

## 4. Statistical Analysis

The data were analyzed via IBM SPSS Statistics 26. Categorical variables are expressed as the number of cases (percentage) (*n* [%]), and continuous variables are expressed as the mean ± standard deviation (*χ* ± *s*). The Kolmogorov–Smirnov test was applied to determine the normality of the quantitative data. Data from the two groups were compared using nonparametric tests. Correlations were analyzed by Spearman correlation analysis, and the strength of the correlation coefficient was measured as follows: *r* < 0.2: no correlation, *r* values from 0.2 to 0.29: weak correlation, *r* values from 0.3 to 0.39: moderate correlation, *r* values from 0.4 to 0.79: strong correlation, and *r* ≥ 0.8: very strong correlation.

## 5. Results

### 5.1. Clinical Characteristics

The male‐to‐female ratio of SMPP children was 1.19:1, and all children had fever. The symptoms of cough and dyspnea in SMPP children significantly differed across ages (*p* < 0.05) and increased mainly in infancy and early childhood. There were 230 children (76.16%) with SMPP who received BAL, and there was a statistically significant difference in BAL demand among the various ages (*p* < 0.05); moreover, the demand for BAL increased significantly among preschool‐aged and school‐aged children. All SMPP children were tested for etiology, of whom 141 (46.69%) were infected by pathogens other than MP. The virus coinfection rate was the highest, followed by bacterial coinfection and fungal coinfection. There was no significant difference in other pathogenic infections among SMPP children of various ages (*p* > 0.05) (Table 1 and Table 2).

**TABLE 1:** Comparison of general data and clinical symptoms across the various ages in the SMPP group (*n* [%]/[±s]).

	Infant group (*n* = 11)	Toddler group (*n* = 31)	Preschool age group (*n* = 114)	School age group (*n* = 146)	*p*
Gender (male) (*n* [%])	6, 54.55	14, 45.16	61, 53.51	83, 56.85	0.693
Days in hospital (days)	14.59 + 6.77	12.71 + 4.99	13.38 + 6.69	14.41 + 6.77	0.440
Days of fever (days)	10.45 + 7.64	10.82 + 5.34	11.15 + 5.86	11.84 + 7.78	0.765
Cough (*n* [%])	11, 100	27, 87.09de	110, 96.49	143, 97.95	0.029
Dyspnea (*n* [%])	6, 54.54bc	11, 35.48e	24, 21.05	27, 18.49	0.012
Chest pain (*n* [%])	0	2, 6.45	6, 5.26	12, 8.22	0.632
BAL (*n* [%])	6, 54.55	18, 58.06	86, 75.44	116, 79.45	0.033

*Note:* b represents the infant group compared to the preschool group (*p* < 0.05), c represents the infant group compared to the school‐age group (*p* < 0.05), d represents the toddler group compared to the preschool group (*p* < 0.05), and e represents the toddler group compared to the school‐age group (*p* < 0.05).

**TABLE 2:** Comparison of pathogen distribution among the various ages of the SMPP group (*n* [%]).

	Infant group (*n* = 11)	Toddler group (*n* = 31)	Preschool age group (*n* = 114)	School age group (*n* = 146)	*p*
Bacteria (*n* [%])	1, 9.09	8, 25.81	23, 20.18	21, 14.38	0.313
Virus (*n* [%])	3, 27.27	8, 25.81	26, 22.81	27, 18.49	0.215
Fungus (*n* [%])	0	3, 9.68	10, 8.77	11, 7.53	0.749

### 5.2. Laboratory Data

There were statistically significant differences in PLT, FIB, D‐D, and CR among SMPP children of various ages (*p* < 0.05). The increase in PLT was mainly in infancy and early childhood. FIB in infancy was significantly lower than that in other age groups, and the D‐D in preschool and school‐aged children was significantly greater than that in infants and toddlers (*p* < 0.05), and the CR was significantly greater in the school‐aged group than in the other age groups, but the mean values of all age groups were within the normal reference value range (Table 3).

**TABLE 3:** Comparison of laboratory indices across the various ages of the SMPP group (±s).

	Infant group (*n* = 11)	Toddler group (*n* = 31)	Preschool age group (*n* = 114)	School age group (*n* = 146)	*p*
WBC (10^9^/L)	11.76 ± 5.84	11.68 ± 10.19	11.20 ± 5.41	11.14 ± 5.19	0.961
PLT (10^9^/L)	446.18 ± 166.83bc	392.90 ± 148.34e	353.33 ± 123.41	339.45 ± 136.95	0.025
CRP (mg/L)	26.52 ± 57.71	18.51 ± 27.13	33.50 ± 43.69	38.72 ± 51.29	0.164
PT (S)	12.39 ± 1.62	12.29 ± 1.33	12.19 ± 1.36	12.48 ± 1.21	0.386
APTT (S)	30.21 ± 5.123	28.83 ± 6.37	30.07 ± 7.14	29.57 ± 9.29	0.906
FIB (g/L)	2.32 ± 0.91abc	3.31 ± 0.74	3.67 ± 1.03	3.73 ± 1.10	0.001
D‐D (ug/mL)	0.93 ± 0.91c	0.98 ± 0.89de	2.44 ± 2.61	2.99 ± 3.73	0.016
ESR (mm/h)	27.40 ± 19.22	44.27 ± 25.66	51.28 ± 30.24	47.40 ± 28.59	0.072
ALT (U/L)	30.50 ± 16.22	33.82 ± 49.81	29.21 ± 36.12	41.39 ± 62.51	0.299
AST (U/L)	52.65 ± 32.99	45.98 ± 41.31	41.14 ± 46.27	41.44 ± 37.89	0.782
LDH (U/L)	426.15 ± 233.17	458.98 ± 240.54	453.38 ± 187.98	465.99 ± 279.17	0.945
CK (U/L)	79.90 ± 105.73	74.35 ± 64.20	8.86 ± 59.28	97.95 ± 103.78	0.327
CK‐MB (U/L)	22.28 ± 16.77	18.34 ± 12.62	23.14 ± 20.70	18.97 ± 10.99	0.181
Urea (mmol/L)	2.78 ± 1.11	2.90 ± 0.84	3.24 ± 1.10	3.35 ± 0.97	0.065
CR (umol/L)	19.47 ± 10.83abc	26.69 ± 7.29e	26.81 ± 6.42f	33.90 ± 7.84	< 0.001
UA (umol/L)	232.80 ± 86.23	209.27 ± 81.14	213.66 ± 90.77	193.65 ± 75.39	0.165
PCT (ng/mL)	0.35 ± 0.56	0.51 ± 1.07	0.52 ± 1.28	0.68 ± 1.98	0.812
IL‐6 (pg/mL)	33.44 ± 68.63	28.74 ± 22.72	96.45 ± 229.66	88.52 ± 278.49	0.638

*Note:* a is the infant group compared to the toddler group (*p* < 0.05), b is the infant group compared to the preschool group (*p* < 0.05), c is the infant group compared to the school‐age group (*p* < 0.05), d is the toddler group compared to the preschool group (*p* < 0.05), e is the toddler group compared to the school‐age group (*p* < 0.05), and f is the preschool group compared to the school‐age group (*p* < 0.05).

Given that the reference ranges of humoral immunity, NE, and LY of healthy children of various ages are quite different, the humoral immunity, NLR, and AGR of children of various ages in the SMPP group and GMPP group were compared and statistically analyzed. The IgM concentration and NLR of preschool‐aged and school‐aged children in the SMPP group were greater than those in the GMPP group, and the NLR was lower than that in the GMPP group (*p* < 0.05). The IgA levels of school‐aged children in the SMPP group were greater than those in the GMPP group, and the IgA levels in C3 and C4 were lower than those in the GMPP group (*p* < 0.05). The C3 of preschool‐aged children in the SMPP group was lower than that in the GMPP group (*p* < 0.05). The AGR in the SMPP group was lower than that in the GMPP group at all ages (*p* < 0.05) (Table 4 and Figure 2).

**TABLE 4:** Comparison of humoral immunity, AGR, and NLR at various ages in the SMPP and GMPP groups (±s).

	Infant group		*p*	Toddler group		*p*	Preschool age group		*p*	School age group		*p*
	SMPP (*n* = 11)	GMPP (*n* = 16)		SMPP (*n* = 31)	GMPP (*n* = 25)		SMPP (*n* = 114)	GMPP (n49)		SMPP (*n* = 146)	GMPP (n43)	
IgG (g/L)	6.97 ± 3.13	8.61 ± 1.77	0.139	8.37 ± 1.76	8.73 ± 1.99	0.530	9.47 ± 3.79	8.43 ± 2.28	0.093	9.63 ± 2.06	8.89 ± 1.81	0.054
IgM (g/L)	1.03 ± 0.30	1.47 ± 0.58	0.058	1.86 ± 1.49	1.30 ± 0.50	0.128	2.08 ± 1.53	1.22 ± 0.49	< 0.001	2.20 ± 1.57	1.16 ± 0.43	< 0.001
IgA (g/L)	0.59 ± 0.28	0.91 ± 0.39	0.060	0.90 ± 0.38	1.16 ± 0.49	0.055	1.21 ± 0.60	1.05 ± 0.70	0.172	1.77 ± 0.79	1.12 ± 0.53	< 0.001
C3 (g/L)	1.08 ± 0.47	1.41 ± 0.51	0.149	1.31 ± 0.38	1.48 ± 0.50	0.209	1.27 ± 0.33	1.28 ± 0.57	0.834	1.16 ± 0.38	1.33 ± 0.47	0.023
C4 (g/L)	0.58 ± 0.82	0.44 ± 0.45	0.620	0.27 ± 0.10	0.43 ± 0.39	0.060	0.41 ± 0.50	0.87 ± 1.90	0.026	0.48 ± 1.00	1.02 ± 1.41	0.013
NLR	1.52 ± 0.84	1.40 ± 0.83	0.723	3.35 ± 5.26	2.32 ± 1.77	0.287	4.23 ± 3.56	2.09 ± 2.49	< 0.001	5.90 ± 4.56	1.86 ± 1.68	< 0.001
AGR	1.73 ± 0.56	2.15 ± 0.33	0.025	1.59 ± 0.45	1.99 ± 0.49	0.003	1.57 ± 0.52	1.88 ± 0.42	0.001	1.47 ± 0.43	1.78 ± 0.40	< 0.001

FIGURE 2Trend chart.

**Figure:**
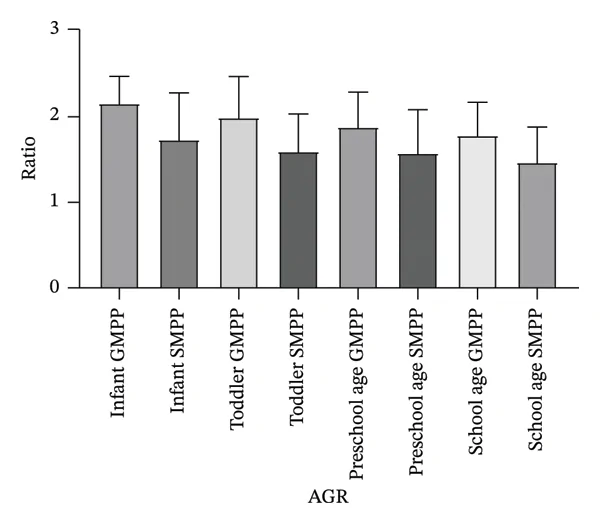
(a)

**Figure:**
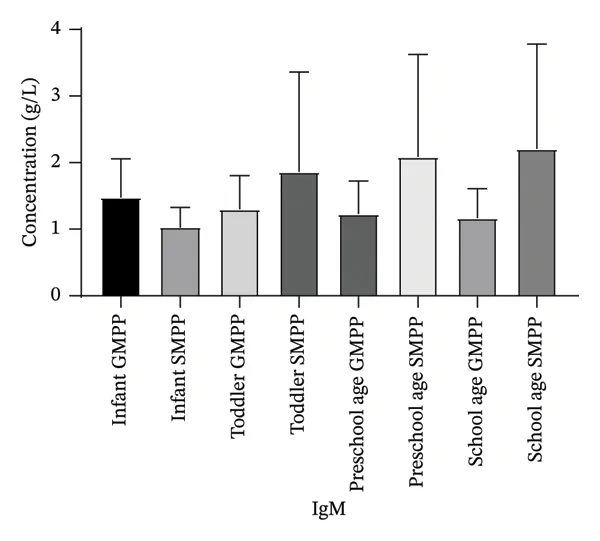
(b)

**Figure:**
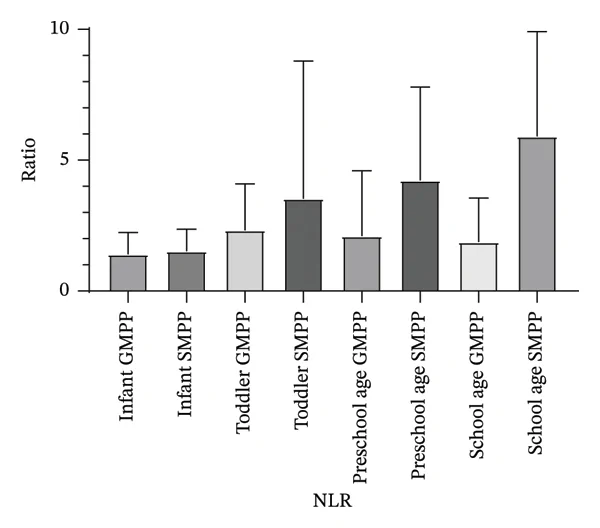
(c)

**Figure:**
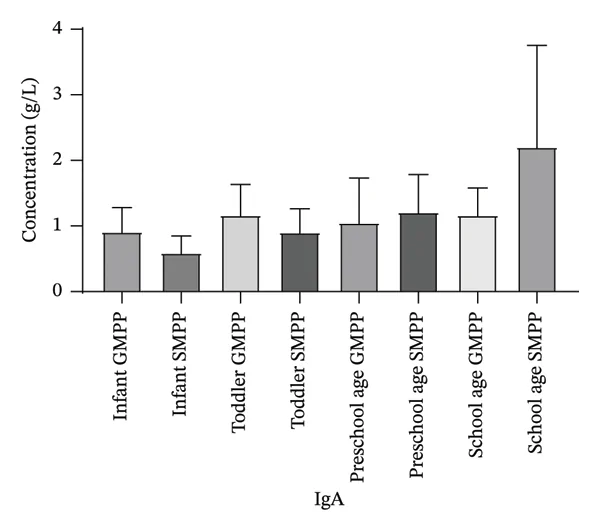
(d)

### 5.3. BAL Microscopic Airway Characterization

A total of 230 children with SMPP underwent BAL, in which changes in the endotracheal and bronchial mucosa and the nature and distribution of secretions could be visually observed. In the SMPP group, there were statistically significant differences in mucosal erosion and mucous plug blockage among children of various ages (*p* < 0.05). Compared to toddlers and preschool children, school‐aged children exhibited a higher rate of mucosal erosion, and compared to toddlers, school‐aged children exhibited a higher rate of mucous plug blockage (*p* < 0.05). (Table 5).

**TABLE 5:** Comparison of bronchoscopic airway changes across the various ages in the SMPP group (*n* [%]).

	Infant group (*n* = 6)	Toddler group (*n* = 18)	Preschool age group (*n* = 86)	School age group (*n* = 116)	*p*
Mucosal congestion and swelling	6	18	86	116	
Mucosal erosion	1, 16.67	1, 5.56e	18, 20.93f	39, 33.62	0.032
Mucosal necrosis	0	0	1, 1.16	3, 2.59	0.787
Mucous plug blockage	0	2, 11.11e	24, 27.91	43, 37.07	0.037
Plastic bronchitis	0	0	8, 9.30	10, 8.62	0.502
Inflammatory narrowing of lobar or segmental bronchial openings	1, 16.67	2, 11.11	7, 8.14	9, 7.76	0.856
Pulmonary hemorrhage	0	0	0	1, 1.17	

*Note:* e represents the toddler group compared to the school‐age group (*p* < 0.05), and f represents the preschool group compared to the school‐age group (*p* < 0.05).

### 5.4. Correlation Ánalysis Between Laboratory Indicators and Mucosal Erosion and Mucous Plug Blockage in Preschool‐Aged and School‐Aged Children With SMPP

In the SMPP group, IgM was positively correlated with mucosal erosion and mucous plug blockage in preschool‐aged and school‐aged children (*r* = 0.341, 0.214, 0.392, and 0.343; *p* < 0.05), and the AGR was negatively correlated with mucosal erosion and mucous plug blockage (*r* = −0.193, −0.217, −0.337, and −0.434; *p* < 0.05). The NLR and D‐D were positively correlated with mucus plug blockage in preschool‐aged and school‐aged children (*r* = 0.189, 0.435, 0.275, and 0.345; *p* < 0.05). IgA and FIB were positively correlated with mucus plug blockage in school‐aged children (*r* = 0.224 and 0.355; *p* < 0.05). FIB was positively correlated with mucosal erosion in school‐aged children (*r* = 0.381; *p* < 0.05).

### 5.5. Discussion

MPP can be seen in all stages of children, especially in preschool‐aged and school‐aged children. The strength of the body’s immune response is closely related to the severity of MPP, which is stronger in older children [7]. Studies have indicated that the median ages of children with MPP are 7 (4.0–11.0) years and 5.1 (4.0–7.9) years [8]. Ding et al. [9] reported that SMPP occurs mainly in preschoolers and that the incidence of SMPP in infants and toddlers is significantly lower than that in other age groups. The median age of the children with SMPP included in the present study was 71 (3–168) months, which is broadly in line with the above studies. The immune function of infants and toddlers is not perfect, and their immune response is weak. The immune system of older children is relatively mature and resistant. When MP invades the body, the immune system is activated, leading to the disease progression of MPP. In addition, this study revealed that the median age in the SMPP group was greater than that in the GMPP group (57 [2–144] months), suggesting that the severity of the disease is related to immune function.

Peripheral blood tests have been used to identify indicators associated with the risk prediction of GMPP and SMPP. The NLR is the ratio of the peripheral blood neutrophil count to the lymphocyte count and reflects the systemic inflammatory response and immune balance [10]. In recent years, the NLR has been used in the diagnosis and severity assessment of MPP [11, 12]. This study revealed that the NLR was significantly greater in preschool‐aged and school‐aged children in the SMPP group than in those in the GMPP group, which was consistent with the findings of the present study, suggesting that after MP infection, the neutrophil count increased because of the anti‐inflammatory response, and the lymphocyte count decreased because of defense, resulting in an increase in the NLR. The NLR is a potential risk factor for SMPP, suggesting that patients with abnormal changes in the NLR should be vigilant against SMPP.

It has been suggested that the precursor cells of the bone marrow in infancy are more responsive to the disease, with increased PLT production in the early stages of the disease, and that PLTs are highly sensitive to the early prediction of the severe development of MPP in infants and toddlers [12, 13]. In the SMPP group in the present study, there were significant differences in the PLT count across the various ages. The PLT in infancy was significantly greater than that in preschool‐aged and school‐aged children, and the PLT in early childhood was significantly greater than that in school‐aged children, suggesting that the difference in PLT count is particularly significant in infancy and early childhood.

Humoral immune dysfunction is common in MPP children, and dynamic monitoring of complement and immunoglobulin is highly valuable for evaluating the severity of the disease. Studies have shown that the increase in IgM in MPP children older than 6 years is likely related to the activation of B cells to secrete immunoglobulin after MP infection, and IgM is involved mainly in the immune response during the acute phase. IgA reaches normal levels in adolescence and is the main antibody involved in local mucosal anti‐infection immunity [14]. Compared to the GMPP group, the SMPP group had higher IgM levels among preschool and school‐aged children; compared to the GMPP group, the SMPP group had higher IgA levels among school‐aged children. This study revealed that there was no statistically significant difference in IgG levels between the SMPP group and the GMPP group at various ages. IgG‐secreting B cells mature earlier and reach human levels at 2 years of age [15]; thus, IgG has little effect on the severity of disease after MP infection.

The complement system plays an important role in eliminating MP‐adherent cells and toxic metabolites and is related to the severity of the disease [16, 17]. Previous studies have reported elevated C3 but not C4 levels in children aged 1–6 years, suggesting that MP may activate complement through the bypass pathway. In contrast, both C3 and C4 are markedly elevated in children older than 6 years, indicating that MP activates complement via the classical pathway after age 6. This study revealed that the C3 level in the SMPP group was lower than that in the GMPP group at preschool age. School age The levels of C3 and C4 in the SMPP group were lower than those in the GMPP group. In the SMPP group, the complement system of the classical pathway was overactivated in preschool‐aged and school‐aged children, with significant consumption of C3 and C4.

The TP used for liver function is composed of albumin and globulin. Globulin is a serum protein produced by the liver and the immune system. Viral antigens can promote the production of immunoglobulin. An increase in globulin can be regarded as a marker of inflammatory activation [18]. Albumin is limited by a number of factors, such as liver synthesis function and nutritional status, which affect its stability in the clinic. The ratio of the serum albumin count to the globulin count (AGR), which effectively avoids the above factors, is a more suitable and accurate prognostic marker. In this study, the AGR in the SMPP group was lower than that in the GMPP group across the various age groups. These findings suggest that an imbalance in immune function and AGR is an effective predictor of SMPP.

Some studies have shown that the abnormal increase in FIB and D‐D in severe pneumonia is beneficial for disease diagnosis and prognosis evaluation, and that FIB and D‐D are significantly greater in MPP children than in healthy children [19, 20]. This study revealed that the FIB of children with SMPP in other age groups was significantly greater than that in the infant age group and that the D‐D in the preschool‐aged and school‐aged groups was significantly greater than that in the infant and toddler age groups. The abnormal increase in FIB and D‐D may be related to the activation of the immune‐inflammatory response in children with SMPP, which leads to abnormal enhancement of coagulation function, and the increase becomes more obvious with increasing age.

Pathogens can trigger an immune response by adhering to mucosal epithelial cells, prompting B cells to secrete antibodies. However, the immature immune system of children is prone to antibody subclass imbalance, leading to defects in mucosal barrier defense and exacerbating tissue damage [21]. Pathogen antigens and antibodies may form immune complexes that deposit in the basement membranes of mucosal blood vessels, resulting in increased vascular permeability, mucosal edema, and necrosis [22]. In BAL, changes in airway mucosa can be observed, whether abnormalities in airway structure can be detected, and the nature and location of secretions in the airway can be clarified [23]. In this study, 230 (76.16%) children with SMPP underwent BAL, and compared with those of infants and toddlers, the incidence of BAL demand, mucosal erosion, and mucus plug blockage increased in preschool‐aged and school‐aged children. Plastic bronchitis, mucosal necrosis, and pulmonary hemorrhage mostly occur in preschool‐aged and school‐aged children.

Previous studies on SMPP have focused primarily on single dimensions: either examining changes in immune function and inflammatory factors or exploring the clinical application and efficacy of BAL. However, no systematic research has yet combined both approaches for early warning and guiding intervention timing. In this study, both dimensions were integrated, and Pearson correlation analysis revealed that IgM was positively correlated with mucosal erosion and mucus plug blockage, AGR was negatively correlated with mucosal erosion and mucus plug blockage, and the NLR and D‐D were positively correlated with mucus plug blockage in preschools and school‐aged patients with SMPP. In school‐aged patients with SMPP, IgA and FIB were positively correlated with mucus plug blockage, and FIB was positively correlated with mucosal erosions.

In conclusion, some clinical manifestations and laboratory hematological indices significantly differ across age groups in SMPP. IgM, IgA, NLR, AGR, FIB, and D‐D are potential markers for assessing disease severity and mucosal damage in preschool‐aged and school‐aged children. Clinical diagnosis should incorporate multiple observational indicators to enable early assessment of MPP severity, thereby more accurately identifying critically ill children and preventing delays in treatment. However, the research scope needs to be expanded, and the sample size should be increased in the future for further in‐depth study.

## Author Contributions

Peng Li and Chunlan Song conceived and designed the study. Peng Li, Wanyu Jia, and Chunlan Song collected and analyzed the data. Peng Li drafted and critically revised the manuscript. Ruiyang Sun, Wanyu Jia, Shuqin Fu, Junhao Cui, Jiapu Hou, and Zewen Ding contributed to administrative, technical, or material support. All authors reviewed the manuscript. All the authors agree to be accountable for the content and conclusions of this article.

## Funding

This study was supported by the Henan Provincial Medical Science and Technology Research Project (China) (LHGJ20240568 and LHGJ20240545) and the Henan Provincial Science and Technology Research Project (China) (252102311119).

## Disclosure

Third‐Party Services Statement. No other persons or third‐party services were involved in the research or manuscript preparation. All the authors agree to be accountable for the content and conclusions of this article.

## Ethics Statement

This study was approved by the Medical Ethics Committee of Henan Children’s Hospital (2025‐KY‐0037‐001). This study is a retrospective study. It has been approved for exemption from signing the informed consent form and has been approved by the ethics committee.

To ensure participant confidentiality, all direct identifiers were removed upon data collection and replaced with a unique study code.

## Consent

Please see the Ethics Statement.

## Conflicts of Interest

The authors declare no conflicts of interest.

## Data Availability

The original contributions presented in the study are included in the article, and further inquiries can be directed to the corresponding author.
